# Reactive Oxygen Species Facilitate Translocation of Hormone Sensitive Lipase to the Lipid Droplet During Lipolysis in Human Differentiated Adipocytes

**DOI:** 10.1371/journal.pone.0034904

**Published:** 2012-04-06

**Authors:** Sarah A. Krawczyk, Jorge F. Haller, Tom Ferrante, Raphael A. Zoeller, Barbara E. Corkey

**Affiliations:** 1 Departments of Medicine and Biochemistry, Boston University School of Medicine, Boston, Massachusetts, United States of America; 2 Department of Physiology and Biophysics, Boston University School of Medicine, Boston, Massachusetts, United States of America; Wayne State University, United States of America

## Abstract

In obesity, there is an increase in reactive oxygen species (ROS) within adipose tissue caused by increases in inflammation and overnutrition. Hormone sensitive lipase (HSL) is part of the canonical lipolytic pathway and critical for complete lipolysis. This study hypothesizes that ROS is a signal that integrates regulation of lipolysis by targeting HSL. Experiments were performed with human differentiated adipocytes from the subcutaneous depot. Antioxidants were employed as a tool to decrease ROS, and it was found that scavenging ROS with diphenyliodonium, N-acetyl cysteine, or resveratrol decreased lipolysis in adipocytes. HSL phosphorylation of a key serine residue, Ser552, as well as translocation of this enzyme from the cytosol to the lipid droplet upon lipolytic stimulation were both abrogated by scavenging ROS. The phosphorylation status of other serine residues on HSL were not affected. These findings are significant because they document that ROS contributes to the physiological regulation of lipolysis via an effect on translocation. Such regulation could be useful in developing new obesity therapies.

## Introduction

Hormone Sensitive Lipase (HSL) is a key enzyme in the regulation of lipid, the largest energy reserve in the body. Recently, there has been a renewed interest in HSL as an attractive therapeutic candidate for obesity because of its crucial role in lipolysis. The lipolytic pathway has been described for quite some time; however, the regulation of this pathway is not as well defined. In this study, we examine regulation of lipolysis and specifically HSL modulation by reactive oxygen species (ROS), which are increased in obesity.

Reduced activity of HSL improves metabolic homeostasis. Mice that lack a functional copy of the gene encoding HSL are resistant to both genetic and diet-induced obesity [1], [2]. Additionally, human studies have revealed that carrying an allele associated with decreased HSL hydrolytic activity is associated with an improved metabolic phenotype. Specifically, women carrying this allele have lower basal and stimulated insulin secretion, and men with this allele have lower circulating non-esterified fatty acids (NEFAs) [3].

As the name suggests, HSL hydrolyzes esters of neutral lipids, principally diacylglyceride (DG), in a manner activated by a variety of hormones that increase cAMP, including catecholamines, adrenocorticotropic hormone (ACTH) and glucagon [4]. Protein Kinase A (PKA), activated via an increase in cAMP, phosphorylates rat HSL on three serine residues, Ser563, Ser659 and Ser660 [5]. These three sites are conserved in human HSL, as Ser552, Ser649 and Ser650, respectively [6]. *In vitro*, phosphorylation of human HSL Ser649 and Ser650 are the major determinants of its hydrolytic activity [7].

Upon phosphorylation, HSL translocates to the lipid droplet to participate in lipolysis. PKA phosphorylation induces a conformational change to expose hydrophobic groups on HSL, which facilitates HSL binding to its substrate, lipid [8]. However, it is unknown which of these three PKA-mediated serine residues is the major determinant of translocation of HSL from the cytosol to the lipid droplet upon lipolytic stimulation. This important layer of regulation also regulates other lipid handling enzymes, including Lipin 1 and ACSL [9], [10].

ROS is a candidate for the regulation of lipolysis, because there is a positive correlation between both ROS and lipolysis with obesity [11]–[14]. In obesity, inflammation and overnutrition converge on an increase in ROS. Recently, there has been a paradigm shift that ROS, previously described as a trigger of programmed cell death and a useless by-product of cellular respiration, is also a signaling molecule and can be helpful rather than exclusively harmful [15]. In fact, ROS has been shown to be a metabolic signal for glucose-stimulated insulin secretion [16]. Given the increase of ROS in obesity and its role as a metabolic signal, we hypothesize that ROS is a modulator of adipocyte lipolysis.

## Results

### ROS Production was Decreased by, Diphenyliodonium (DPI), N-acetyl Cysteine (NAC) and Resveratrol

ROS levels are increased in obesity and decreased by ROS scavengers.

**Figure 1:**
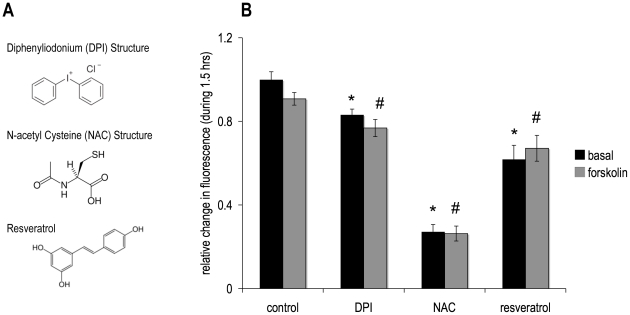
Antioxidant Structures and effect on ROS generation. **(A)** Each antioxidant scavenges ROS as a consequence of their chemical structures. Each also has specific additional mechanisms: Diphenyliodonium (DP) inhibits NADPH oxidase, NAC increases cellular glutathione levels and resveratrol is a sirtuin activator. **(B)** Human differentiated adipocytes were preincubated for half an hour in DMEM with 1% BSA and 5 µM of the dye, CM-H_2_DCFDA. Cells were then washed and media were changed to HBSS. Compounds were added (10 µM DPI, 5 mM NAC or100 µM resveratrol) in the absence or presence of 5 µM forskolin and fluorescent readings were taken for 1.5 hours at 37°C at wavelengths 485 nm excitation, 538 nm emission. Data are expressed as the relative change of the final value minus the initial baseline reading. The panel shows normalized mean values ± S.E.M. for three independent experiments; *p <0.05 vs. basal control, # p<0.05 vs. forskolin control.

It can be a challenge to increase ROS levels modestly in cell culture models, although scavenging with antioxidants is feasible. Various reactive species have different half-lives and may act in different compartments that are difficult to target in a cell culture system. Also, a portion of ROS is scavenged by components of the experimental media including pyruvate [17]. Thus, many experimental designs use superphysiological ROS treatments, which may not be physiologically relevant. For these reasons, our studies used several antioxidants to decrease ROS levels in cultured adipocytes. To validate the decrease in ROS levels in this model, cells were incubated with established antioxidants [18]–[20], at concentrations comparable to those used in the literature of ROS-related research, and then ROS levels were assessed using the ROS-sensitive intracellular fluorescent dye CM-H_2_-DCFDA. **Figure 1** shows that over 1.5 hours of measurement, ROS levels decreased compared to control with each antioxidant, DPI, NAC and resveratrol. 5 µM forskolin, used in this study to induce lipolysis, had no effect on induction of ROS under control or antioxidant conditions.

### Scavenging ROS Decreased Lipolysis

**Figure 2:**
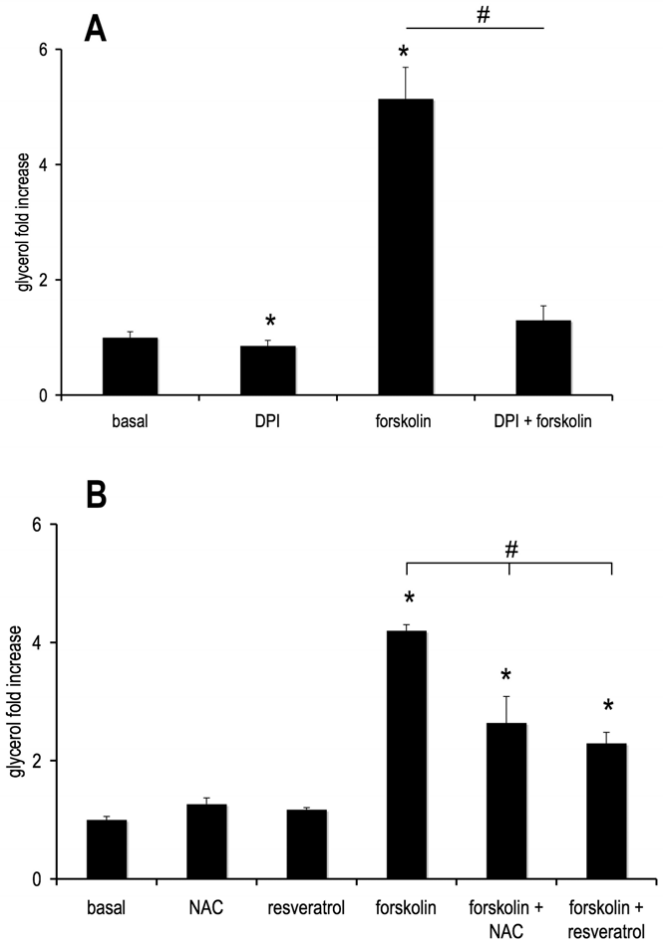
DPI, NAC and resveratrol decrease lipolysis. Human differentiated adipocytes were treated with or without **(A)** 10 µM DPI, **(B)** 5 mM NAC, or 100 µM resveratrol in the absence or presence of 5 µM forskolin for 4 hours in KRB containing 1% BSA and 0.5 mM oleate. Media were collected and assayed for glycerol using an enzyme-linked luciferase assay. Results for glycerol are normalized to the basal condition and represented as fold increase. Average basal value was 16.7 ± 1.7 nmols per million cells per hour, average forskolin-stimulated value was 85.8 ± 9.1 nmols per million cells per hour. Values ± S.E.M. for three independent experiments; * p <0.05 vs. basal control, # p<0.05 vs. forskolin control.

We next assessed lipolysis by measuring glycerol release from cells into the cell culture media. **Figure 2A** shows that in cultured adipocytes, the antioxidant DPI decreased both basal and forskolin-stimulated lipolysis compared to their respective controls. These findings are in agreement with published literature using freshly isolated rat adipocytes [21].

Next, we determined whether the effect of DPI on lipolysis was specific for DPI or if other antioxidants also decreased lipolysis. **Figure 2B** shows that the antioxidants, NAC and resveratrol also inhibited stimulated lipolysis.

### Scavenging ROS did not Alter cAMP, PKA and Perilipin

**Figure 3:**
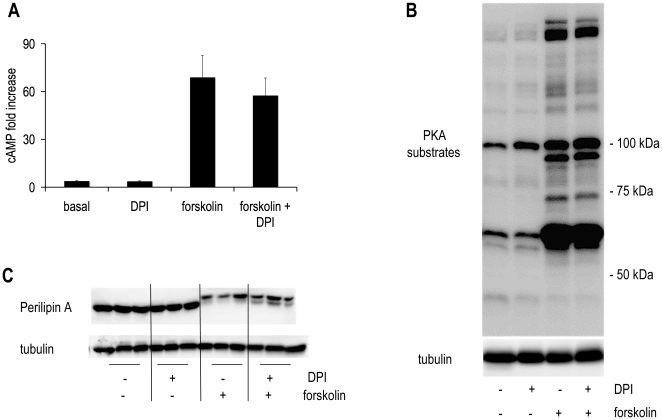
The lipolytic pathway is unaltered by DPI treatment. Human differentiated adipocytes were treated with or without 10 µM DPI in the absence or presence of 5 µM forskolin. **(A)** 15 minutes in KRB with 1% BSA and 0.5 mM oleate. Cell lysates were collected and assayed for cAMP using an ELISA-based assay. **(B, C)** Cells were treated under the same conditions for 4 hours. Individual proteins were analyzed by western blotting with specific antibodies. Signals were visualized with an enhanced chemiluminescence substrate kit (Thermo Scientific, Rockford IL) as described in materials and methods. Results for cAMP are normalized to the basal condition and represented as fold increase. Average basal value was 3.9 ± 0.5 pmols per million cells, average forskolin-stimulated value was 68.9 ± 13.9 pmols per million cells. Values ± S.E.M. for three independent experiments. Immunoblots shown are representative of those obtained in three separate experiments.

Taken together, the above data showed that scavenging ROS inhibited lipolysis. To determine the mechanism, we investigated the cAMP lipolytic pathway. cAMP is a common point of regulation of lipolysis [22]. Upon lipolytic stimulation, cAMP levels peak at 15 minutes [23]. Basal and forskolin-stimulated cAMP levels were assessed at this time point and each was not altered by DPI treatment compared to its respective control (**Figure 3A**). Next, the activity of PKA, cAMP’s downstream target, was assessed using an antibody against an epitope tag for sites phosphorylated by PKA. The western blot in **Figure 3B** shows no alteration of PKA activation under either basal or forskolin-stimulated conditions in the presence of DPI compared to control.

To confirm the lack of effect on PKA, we next measured the phosphorylation state of individual proteins that PKA phosphorylates. Perilipin, a lipid droplet coating protein, is hyperphosphorylated upon lipolytic stimulation. Perilipin phosphorylation facilitates HSL-mediated lipolysis by its interaction with lipid droplet associated HSL [24]. This hyperphosphorylation decreases the mobility of perilipin through an SDS page gel [25]. When a 10% polyacrylamide denaturing gel is run so that lower molecular weight proteins run off the gel, the area around 57 kDa is expanded so that decreased mobility of perilipin can be visualized. In **Figure 3C** perilipin western blots show that although mobility of perilipin after forskolin stimulation was decreased compared to control, DPI did not alter perilipins gel migration pattern in the presence or absence of forskolin stimulation.

**Figure 4:**
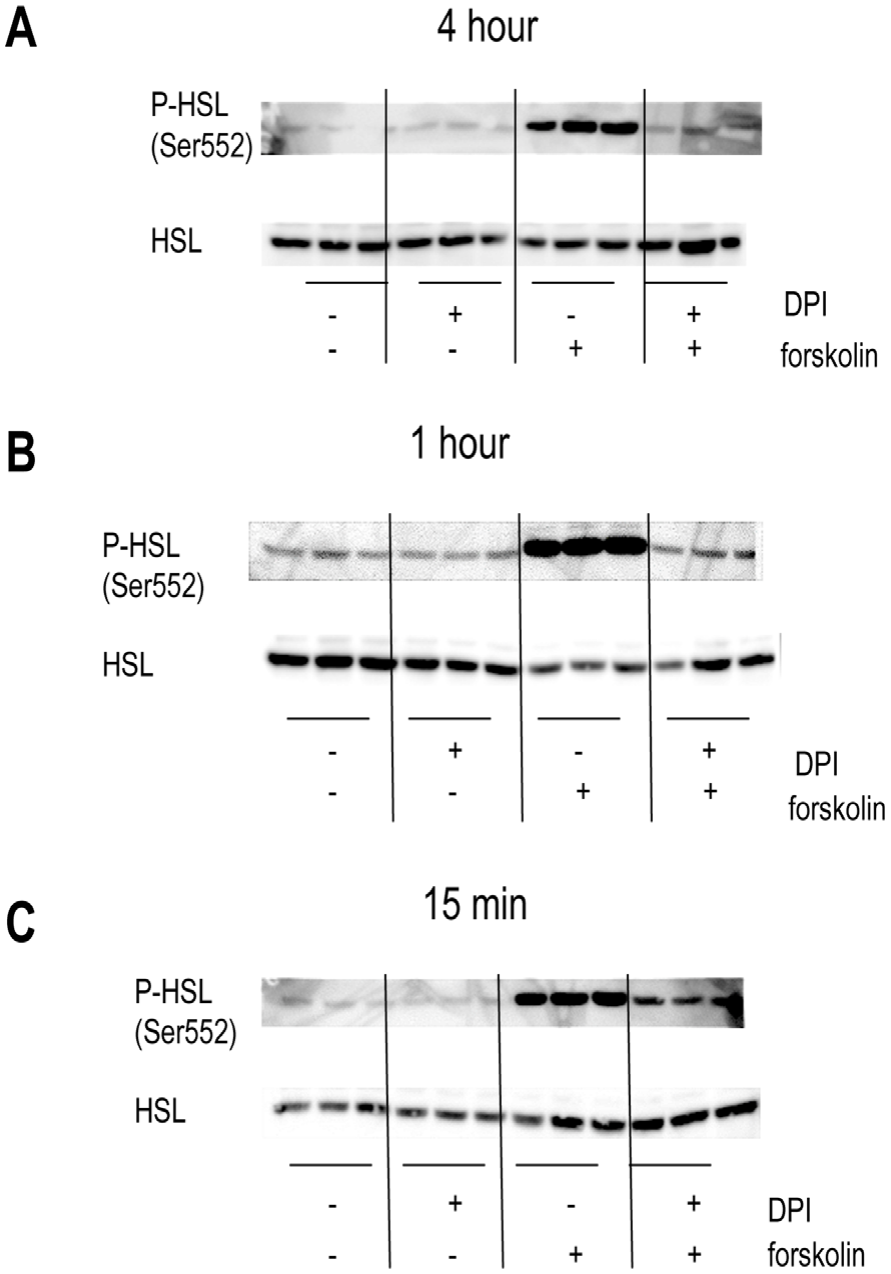
HSL phosphorylation was decreased by DPI treatment under lipolytic stimulation after 15 min, 1 hour and 4 hours. Human differentiated adipocytes were treated with or without 10 µM DPI in the absence or presence of 5 µM forskolin for **(A)** 4 hours **(B)** 1 hour or **(C)** 15 min in KRB with 1% BSA and 0.5 mM oleate. Individual proteins were analyzed by western blotting with specific antibodies. Signals were visualized with an enhanced chemiluminescence substrate kit (Thermo Scientific, Rockford IL) as described in materials and methods. Immunoblots shown are representative of those obtained from three separate experiments.

Hormone sensitive lipase is another target of PKA, though it can also be activated by other kinases [26]. A 4 hour treatment with DPI diminished phosphorylation of HSL on Ser552 (**Figure 4A**). This is a site considered to be a PKA phosphorylation site [3].

Further studies were undertaken to determine how deficient phosphorylation of HSL Ser552 decreased lipolysis in the presence of DPI. We next assessed the time course of decrease in this site’s phosphorylation in the presence of DPI under forskolin stimulation. Phosphorylation of HSL Ser552 was prevented by DPI as early as 15 minutes following compound exposure (our earliest time point) and was maintained at 1 hour of treatment (**Figure 4B and C**).

### HSL Ser554 and Ser650 Phosphorylation was Unaltered by DPI

**Figure 5:**
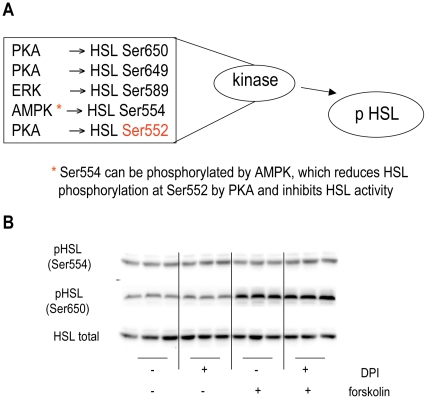
Regulation of HSL by phosphorylation. As reviewed by Lampidonis et al [46]. **(A)** HSL can be phosphorylated by a number of kinases. PKA is able to phosphorylate human HSL at Ser563, Ser649 and Ser650. ERK can phosphorylate HSL at Ser589. AMPK can phosphorylate HSL at Ser554, which in some tissues reduces HSL phosphorylation at Ser552 by PKA and inhibits HSL activity. Phosphorylation at HSL Ser650 and Ser554 is unaltered by DPI treatment. **(B)** Human differentiated adipocytes were treated with or without 10 µM DPI in the absence or presence of 5 µM forskolin for 15 minutes in KRB with 1% BSA and 0.5 mM oleate. Individual proteins were analyzed by western blotting with specific antibodies. Signals were visualized with an enhanced chemiluminescence substrate kit (Thermo Scientific, Rockford IL) as described in materials and methods. Immunoblots shown are representative of those obtained from three separate experiments.

**Figure 5A** summarizes potential mechanisms by which phosphorylation of HSL Ser552 can occur. Studies of mouse HSL reveal that when the neighboring HSL Ser565 (human Ser554), is phosphorylated by AMPK, mouse Ser563 (human Ser552) cannot be phosphorylated [27], [28]. Western blots were probed with anti-pHSL Ser554 antibody and revealed that phosphorylation of this serine was not only unaltered in human differentiated adipocytes by forskolin-induced lipolysis but additionally, the level of phosphorylation was unaltered by DPI (**Figure 5B**). This rules out the possibility of AMPK phosphorylation of HSL Ser552 being important in the context of this study.

Although cAMP levels and PKA substrate phosphorylation of most proteins suggested that there was no alteration in activation of this pathway in the presence of DPI, it is possible that HSL phosphorylation by PKA was blocked even though PKA activity was intact. HSL has been shown to associate with the lipid binding protein aP2 [29] and perhaps this complex cannot be phosphorylated by PKA in the presence of DPI. To address this possibility, experiments were performed that probed western blots with anti-HSL at Ser650, another site that PKA has been shown to phosphorylate [30]. **Figure 5B** shows that HSL Ser650 phosphorylation was increased by forskolin as expected; however this increase in phosphorylation with forskolin-stimulation was unaltered in the presence of DPI. This result suggested that the lack of HSL Ser552 phosphorylation might not be due to either impairment of PKA activity or lack of access of PKA to HSL because HSL Ser660 retained its ability to be phosphorylated under lipolytic conditions in the presence of DPI.

### DPI, NAC and Resveratrol Decreased Forskolin-mediated Translocation of HSL from the Cytosol to the Lipid Droplet

**Figure 6:**
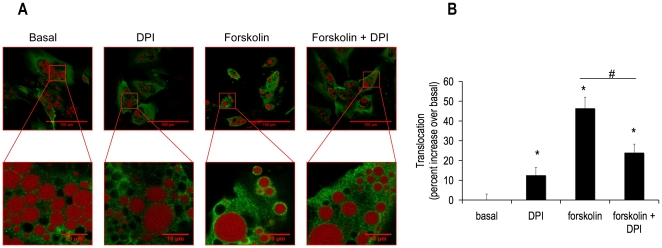
DPI abrogated forskolin-induced HSL translocation from the cytosol to the lipid droplet. Human differentiated adipocytes were grown and differentiated in 35 mm coated glass bottom dishes and then treated with or without 10 µM DPI in the absence or presence of 5 µM forskolin for 15 min in KRB containing 1% BSA and 0.5 mM oleate. Cells were then fixed with paraformaldehyde and subsequently incubated with anti-total HSL antibody (green), cells were also labeled with Lipotox red for lipid droplet detection (red), as described in the methods section. Cells were imaged in these 35 mm dishes in an inverted microscope. Confocal pictures were taken **(A)** Images were analyzed using ImageJ software. **(B)** The panel shows normalized mean values ± S.E.M. for three independent experiments with at least 50 cells per condition per experiment; * p <0.05 vs. basal, # p<0.05 vs. forskolin.

**Figure 7:**
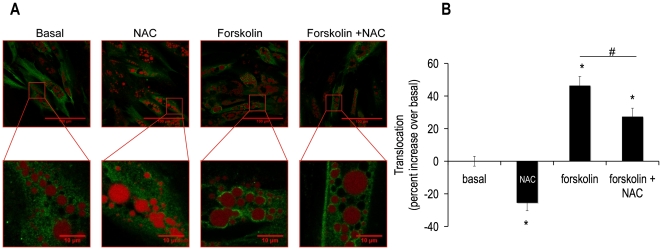
NAC abrogated forskolin-induced HSL translocation from the cytosol to the lipid droplet. Human differentiated adipocytes were treated as described in figure 6 except with or without 5 mM NAC for 15 minutes. Cell were then fixed with paraformaldehyde and subsequently incubated with anti-total HSL antibody (green); cells were also labeled with Lipotox red for lipid droplet detection (red). Confocal pictures were taken **(A)** and analyzed using image J software. **(B)** The panel shows normalized mean values ± S.E.M. for three independent experiments with at least 50 cells per condition per experiment; * p <0.05 vs. basal, # p<0.05 vs. forskolin.

**Figure 8:**
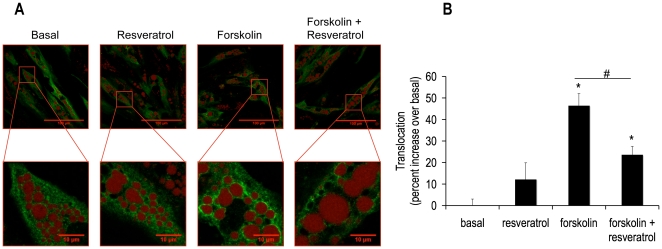
Resveratrol abrogated forskolin-induced HSL translocation from the cytosol to the lipid droplet. Human differentiated adipocytes were treated as described in figure 6 except with or without 100 µM resveratrol for 15 minutes. Cell were then fixed with paraformaldehyde and subsequently incubated with anti-total HSL antibody (green), cells were also labeled with Lipotox red for lipid droplet detection (red). Confocal pictures were taken **(A)** and analyzed using image J software. **(B)** The panel shows normalized mean values ± S.E.M. for three independent experiments with at least 50 cells per condition per experiment; * p <0.05 vs. basal, # p<0.05 vs. forskolin.

We next asked was how an alteration in phosphorylation of HSL Ser552 might translate into a decrease in lipolysis? Mutational analysis reveals that phosphorylation of Ser650 is a major determinant of the hydrolytic activity of HSL, while phosphorylation of Ser552 is not a major contributor [7]. Additionally, phosphorylation of traditional PKA sites (Ser552, Ser649 and Ser650) induces a conformational change of HSL, revealing hydrophobic groups, which may aid in the translocation of this lipase to the lipid droplet [8]. Thus, we asked if our western blot results i.e. a loss of phosphorylation of HSL Ser552 with retained phosphorylation of HSL Ser650 correlated with a decrease in translocation of HSL from the cytosol to the lipid droplet. Fixed cells were examined by confocal microscopy after a 15 minute treatment with DPI. **Figure 6A** shows images demonstrating that DPI decreased forskolin-stimulated translocation of HSL from the cytosol to the lipid droplet. Image analysis of these data documented a 50% decrease in HSL translocation (Fig 6B insert). To determine if this mechanism of lipolytic inhibition was common to all antioxidants tested in these lipolysis experiments, further translocation experiments were performed with NAC and resveratrol treatments. **Figures 7A and 7B** and **8A and 8B** illustrate and quantitate that NAC and resveratrol also inhibited forskolin-stimulated translocation of HSL from the cytosol to the lipid droplet. Taken together, these data suggest a model in which HSL phosphorylation of Ser552 and translocation to the lipid droplet are impaired by scavenging ROS.

## Discussion

### Lipolysis is Altered by Scavenging ROS

The exciting finding from these studies is documentation of ROS as an important regulator of lipolysis. The implication of these data is that: 1) ROS has a physiological role on lipolysis. 2) lipolytic alterations that occur during obesity may be due to ROS. With respect to the physiological role of ROS, the results of the antioxidant studies suggest that ROS is a necessary component of the lipolytic signaling cascade and that when ROS is scavenged by DPI, both basal and stimulated lipolysis are decreased. The antioxidants NAC and resveratrol each also inhibited stimulated lipolysis, suggesting that this was a bona fide antioxidant effect. Experiments performed with freshly isolated rat adipocytes are in agreement with the findings of this study [21].

### Mechanistic Studies of Lipolysis

A number of mechanistic possibilities were assessed to understand how ROS exerted an effect on lipolysis. The lipolytic pathway was unaltered by DPI treatment as evidenced by no alteration in cAMP, PKA substrate phosphorylation, or perilipin phosphorylation. This suggested that DPI inhibited lipolysis in an unconventional manner. One future direction is to assess the cGMP non-canonical lipolytic pathway. Natriuretic peptides (NPs), atrial (ANP) and brain NP stimulate lipolysis *in vitro* [31], [32] and *in vivo* [33], [34] via a cGMP non-canonical lipolytic pathway. Interestingly, HSL is phosphorylated in response to ANP, although the specific serine residue(s) on HSL that are phosphorylated are unknown. Of note, the reactive oxygen species, NO also activates this pathway [35]–[37].

An examination of the DG lipase HSL led to some surprising and novel modes of regulation of lipolysis. The phosphorylation of the PKA site, HSL Ser552 was reduced in the presence of DPI. Since this effect was seen as early as 15 min, it is likely not due to an alteration in either translation or degradation of HSL protein. However, phosphorylation of the PKA target site HSL Ser650 did not change under these same experimental conditions. AMPK is not implicated in the abrogation of HSL Ser552 via increased phosphorylation of HSL Ser554, as phosphorylation of the latter site was unchanged in the presence of DPI. Of note, stimulation of lipolysis, which has been shown to activate AMPK in 3T3-L1 cells [38] does not cause phosphorylation of the AMPK site on HSL, Ser554 in human differentiated adipocytes. Thus, PKA activity was intact in the presence of DPI and phosphorylation of one PKA phosphorylation site (Ser650) was intact.

These findings certainly do not rule out the regulation of other lipases by antioxidants. Adipocyte triglyceride lipase (ATGL) is a candidate for future studies since this lipase is the rate determining enzyme of lipolysis [39] and β-adrenergic activation of ATGL is required for maximal lipolysis [40].

### Translocation of HSL

Work from the laboratory of Cecilia Holm et al revealed that *in vitro*, HSL Ser649 and Ser650 are the major determinants of hydrolytic activity, but not Ser552 [7]. This prompted us to ask if perhaps in humans, Ser552 determines translocation instead of activity of HSL. The results presented here indicate that HSL Ser552 may be involved in translocation of HSL in human adipocytes. The key experiment for this finding was the decrease in translocation of HSL upon forskolin-stimulation in the presence of DPI, a condition where HSL Ser552 phosphorylation was also abrogated and lipolysis was decreased. In 3T3L1 cells, it has been shown that the HSL double mutant of Ser659→Ala/Ser660→Ala (human Ser649 and Ser660) does not translocate to the lipid droplet although single mutations of each PKA site retain the ability to translocate [41].

These data suggest that the ability of the enzyme to be localized near its substrate is an important layer of regulation. To date, this is the first study implicating human HSL Ser552 as a key residue for lipolysis. Evidence in 3T3L1 cells suggests that translocation precedes phosphorylation at HSL Ser563 (human HSL Ser552) [42]. Further experiments need to be performed to determine the order of these two events in human adipocytes.

This is also the first study to suggest that under some conditions, such as altered ROS, two PKA-target phosphorylation sites (HSL Ser650 and Ser552) do not always have the same pattern of phosphorylation. This could suggest that two distinct kinases phosphorylate these two serine residues. Older literature names glycogen synthase kinase 4 (GSK-4) (now renamed to GSK-3ß) as a kinase that phosphorylates HSL at Ser552 [26], [43], [44].

### Conclusions and Unresolved Questions

The testable model that evolves from these data is that ROS is an important signal that promotes lipolysis, and we propose that the mechanism by which this occurs is by altered translocation of HSL from the cytosol to the lipid droplet. This leads to the questions: 1) Under conditions of high ROS, as in obesity, is the increase in lipolysis mediated by ROS? 2) Could other lipolytic components and also lipid synthetic enzymes be regulated in a similar manner by ROS, i.e., translocation? Lastly, this paper aims to instill a renewed interest in HSL as a target of small molecule inhibitors and activators as a means of modulating lipid turnover under conditions of both excess and also reduced lipid storage.

## Methods

### Cell Culture

The differentiation of human primary preadipocytes into adipocytes was carried out essentially as previously described [45]. The Adipocyte Core of the Boston Nutrition Obesity Research Center (BNORC) DK46200 provided the preadipocytes. The cells were completely de-identified and were given to us in a manner that does not link the samples to any private/protected information. Originally, subcutaneous fat tissue was obtained from subjects undergoing panniculectomy surgery by BNORC. The preadipocyte population for five subjects cells were then grown and pooled and the combined subjects cells were seeded at confluence in experimental plates to be differentiated. Average subject was female age 41.6±5.4 with a BMI of 36±7.6. Once differentiated, these 5 subjects’ cells performed according to established standards with respect to response to lipolytic agonists and the rate of neutral lipid synthesis. All experiments were performed with these cells between days 12–14 after differentiation.

### Antibodies

The following antibodies were purchased from Cell Signaling Technology (Beverly, MA): polyclonal antibody against HSL, polyclonal antibody against phosphorylated human HSL serine residue 552 (rat 563), polyclonal antibody against phosphorylated human HSL serine residue 554 (rat 565), polyclonal antibody against phosphorylated human HSL serine residue 650 (rat 660), monoclonal antibody against PKA substrates, and monoclonal antibody against tubulin. Polyclonal antibody against perilipin A was a kind gift from Dr. A. Greenberg (Tufts University), previously described [43].

### Lipolysis Assay

Cells were preincubated in Krebs Ringer Buffer (KRB) (130 mM NaCl, 4.7 mM KCl, 2.5 MgSO_4_ mM, 3.3 mM CaCl_2_, 24.5 mM NaHCO_3_, 1 mM KH_2_PO_4_, 5 mM glucose, incubated for 10–15 min with 5% CO_2_, 95% O_2_) for 30 minutes and then switched to KRB with 0.5 mM oleate and 150 µM BSA in the presence or in the absence of 5 µM forskolin for 4 hours. Glycerol release was measured by using a luciferase based enzyme-linked luminescent assay previously described [44]. All reagents and enzymes were purchased from Roche (Indianapolis, IN). Antioxidants were validated to not cause an artifact in this assay by adding each to a standard curve and observing that there was no difference in each standard curves with or without antioxidant.

### Immunoblotting

Proteins were separated in 10% SDS-polyacrylamide gels (Biorad, Hercules CA) and transferred to a polyvinylidene fluoride 0.2 µm membrane (Biorad). Membranes were blocked with 5% nonfat milk in TBS-T buffer (150 mM NaCl, 20 mM Tris-HCl, pH 7.4, 0.1% Tween-20) for 1 hour. Membranes were then probed overnight at 4°C with primary antibodies followed by 1 hour incubation with secondary antibody conjugated to horseradish peroxidase. Protein bands were detected with an enhanced chemiluminescence substrate kit (Thermo Scientific, Rockford IL) using a FujiFilm LAS-4000 Luminescent Image Analyzer.

### cAMP Measurement

cAMP was measured using a fluorescent assay kit (Molecular Devices, Sunnyvale, CA) according to manufacturer’s instructions.

### ROS Measurement

ROS measurements were performed with the ROS sensitive dye CM-H_2_DCFDA, which cleaves to become DCF (Invitrogen Molecular Probes, Eugene OR). Cells were grown and differentiated in 12-well plates (2.3 × 10∧^5^ cells per well). On the day of the experiment, media was changed to DMEM with 1% BSA. CM-H_2_DCFDA was added for a 30 minute preincubation at a final concentration of 5 µM. Cells were then washed and media was changed to Hank’s Balanced Salts (HBSS). Fluorescent readings were taken on a plate reader (Tecan, Austria) every 30 seconds for 1.5 hours at 37°C at excitation 485, emission 538 nm. A preliminary experiment was performed to establish conditions and it was determined that BSA interferes with the assay and was therefore not included during the 1.5 hours of measurement although 0.1% is added during the loading of CM-H_2_DCFDA into the cells.

### Immunocytochemistry

Cells were grown and differentiated in 35 mm coated glass bottom dishes (Greiner bio-one, Advanced glass bottom Germany) and subsequently imaged in these plates in an inverted microscope. At the commencement of the experiment, cells were fixed for 20 minutes in 4% formaldehyde and then blocked and concomitantly permeabilized for one hour in PBS with 0.3% Triton X-100 and 5% goat serum at room temperature. Cells were stained overnight at 4°C with the primary antibody, anti-HSL (Cell Signaling, Beverly, MA) at a 1∶200 dilution in PBS with 0.3% Triton X-100 and 1% BSA, followed by a one hour incubation at room temperature with Alexa Fluor 488 anti-rabbit secondary antibody (Invitrogen, Eugene, OR) at a 1∶400 dilution, while concomitantly staining with Lipotox red stain (Invitrogen, Eugene, OR) at a 1∶1000 dilution to visualize lipid droplets and Hoescht 33342 (AnaSpec Inc, San Jose, CA) at a 1∶1000 dilution to visualize nuclei. The solution was then replaced with 100 µl of SlowFade Gold antifade reagent (Invitrogen, Carlsbad, CA) and cells were examined by confocal fluorescent microscopy using a Zeiss LSM 710-Live Duo scan microscope. (Zeiss, Thornwood, NY).

### Confocal Image Analysis

Data were analyzed using ImageJ software from the National Institute of Health (NIH) (Bethesda, MD) and can be found at http://imagej.nih.gov/ij/. **Figure S1** is the macro that was generated and run in ImageJ to analyze HSL translocation from the cytosol to lipid droplets. Briefly, the goal was to measure the pixel intensity around each lipid droplet and also measure the pixel intensity within the cytosol. This ratio of average pixel intensity around the lipid droplet over average pixel intensity of the cytosol was termed the ‘translocation to the lipid droplet’ and was used as an index to compare experimental conditions to control.

### Data Analysis and Statistics

Student’s paired two-tailed t test was used to evaluate statistical significance of results. Values of p < 0.05 were considered to be significant differences.

## Supporting Information

Figure S1 **Macro to analyze HSL translocation with Image J software.** For the pixel intensity around the lipid droplet, the confocal picture of the lipid droplets, detected using Lipotox red stain, was converted into a mask. This mask was then duplicated and one copy was dilated twice. The unaltered lipid mask was then subtracted from the twice dilated lipid mask and the resulting mask outlined the perimeters of the lipid droplets. This ‘peri-lipid’ mask was then applied to the HSL confocal picture and the number of pixels and intensity at each pixel could be extracted from the histogram for this picture. For the cytosolic pixel intensity, a mask was generated from the HSL confocal picture. This mask was then subtracted from the twice dilated lipid mask generated for the ‘peri-lipid’ analysis. The resulting mask, termed ‘HSL mask’, included all of the cytosol except that which was directly around each lipid droplet. This HSL mask was then applied to the HSL confocal picture and the number of pixels and intensity at each pixel could be extracted from the histogram for this picture. The index ‘translocation to the lipid droplet’ was then calculated using the average pixel intensities from the peri-lipid and cytosol analyses.
(TIF)
